# A Two-Dimensional Perspective on Quality Of Life: Changes After the Balancing Everyday Life Intervention for Mental Health Service Users and Relationships to Everyday Occupations – An RCT Study

**DOI:** 10.1007/s44474-026-00013-3

**Published:** 2026-11-13

**Authors:** Mona Eklund, Elisabeth Argentzell

**Affiliations:** Department of Health Sciences, Lund University, Lund, Sweden

**Keywords:** Activity, Occupational therapy, Psychiatry, Recovery, Satisfaction, Well-being, Work

## Abstract

**Purpose:**

A randomized controlled trial (RCT) evaluating the Balancing Everyday Life (BEL) intervention compared with care as usual (CAU) for mental health service users found no group differences when quality of life (QoL) was treated as a single construct. However, research suggests that (QoL) consists of two dimensions: a personalized life-and-health dimension (e.g., life satisfaction, health, leisure, and work) and an environmental dimension (e.g., safety, housing, and family situation). The current study re-examined data from the BEL study to investigate whether the BEL group and the CAU differed regarding improvements on the two aforementioned QoL dimensions and explore how changes in everyday occupations were related to QoL improvements.

**Methods:**

A total of 226 mental health service users were assigned to BEL or CAU. Data were collected at baseline, post-intervention, and six-month follow-up. QoL was measured using the Manchester Short Assessment of Quality of Life (MANSA) and analyzed as two dimensions. Everyday occupations were assessed through measures of actual participation and subjective experiences. Change scores were analyzed using t-tests and Pearson correlations.

**Results:**

The BEL group showed significantly greater improvement in the life-and-health dimension of QoL after the intervention, with marginal significance at follow-up. Positive perceptions of everyday occupations were moderately associated with improvements in both QoL dimensions, whereas actual activity participation showed weaker associations.

**Conclusion:**

Treating QoL as a two-dimensional construct revealed intervention effects that were not detected in previous analyses. Interventions emphasizing meaningful everyday occupations may enhance QoL and recovery among mental health service users. Trial Registration The study was registered with ClinicalTrial.gov. Reg. No. NCT02619318. Retrospectively registered: 20-11-2015.

## Introduction

Quality of life and related concepts, such as well-being and personal recovery, have taken a central role in mental health intervention research over the past few decades [1]. Generally, quality of life measurements result in a global score, but researchers have proposed that quality of life is best understood as composed of more than one factor, where one is more oriented towards the personal sphere and the other relates to the environmental [2, 3]. Satisfaction with life as a whole, physical and mental health, leisure, job, and one’s financial situation are examples of what forms a life-and-health aspect of quality of life. Interpersonal, social, and societal factors in one’s environment compose an environmental aspect, where satisfaction with safety, accommodation and one’s family situation are contributing factors [2]. Both aspects have been found to be associated with satisfaction with everyday occupations among people with severe mental illness [3], but less is known regarding how the aforementioned quality-of-life aspects are related to other aspects of everyday occupations, such as actual doing. In the context of mental health interventions, gaining a detailed understanding of the relationships between everyday occupations and quality of life can improve the knowledge base regarding recovery among people with severe mental illness and how to support them towards a more satisfying life quality. Even more importantly, when it comes to the investigation of intervention outcomes, improvements in one quality of life aspect may not be accompanied by improvements in the other, and such discrepancies may go unnoticed if all items are aggregated into a single overall quality of life score. These knowledge gaps served as the rationale for the current study, which is a re-analysis of data from an RCT evaluating a group- and activity-based intervention for people with severe mental illness, termed Balancing Everyday Life (BEL), where quality of life was one of the intervention outcomes [4].

The group- and recovery-based BEL intervention [4] was developed for people with severe mental illness in need of better balanced and more satisfying everyday occupations, including work-related occupations, home chores, personal care, leisure, social activities, etc. Co-produced with a panel of research-educated service users, the 16-week program targets themes related to everyday occupations and supports participants in finding motivation and identifying meaningful daily occupations [5]. Themes such as “how to feel productive” or “ways of finding my motivation” are discussed in the group, followed by setting a personal activity goal and trying to carry it out. The outcome is then discussed in the next group session, and the resulting group cohesion and support have been found to be crucial for making changes toward a more balanced and meaningful lifestyle [6]. Compared with care as usual (CAU), the intervention proved effective in enhancing occupational engagement and psychosocial functioning between baseline and completion [4]. That latter study did not indicate any difference in quality of life improvement, assessed as a sum score of satisfaction with various life domains [7], between the intervention arms from baseline to completed intervention, but the group receiving the BEL intervention had improved more than the CAU group at a 6-month follow-up [4]. That study did not distinguish between a personalized life-and-health and an environmental aspect of quality of life, however, nor did it address how specific aspects of everyday occupations were related to the quality of life outcomes. This warrants further analysis of the quality of life data from that RCT.

In the study of everyday occupations as means and outcomes in interventions for people with mental health service users, researchers have found it relevant to address the actual doing – the performance – as well as the personal experience the performance brings to the doer, in terms of satisfaction, meaningfulness, and/or value [4, 8–10]. Both aspects are central to the BEL intervention, which emphasizes not only following through on one’s occupational goals but also the satisfaction and sense of meaning associated with the doing. Increased knowledge about how changes in various aspects of actual doings (such as activity level and work-related factor) and personal experiences (such as occupational satisfaction) are related with changes in two facets of quality of life—a personalized life and health aspect and a satisfaction with living environment aspect [2]—seems important. Hypothetically, improvements in one aspect of quality of life may not coincide with improvements in another and discrepancies may remain undetected when all items are aggregated into a single overall score. The more detailed insight gained by distinguishing between the two facets could reveal which interventions benefit which aspect of quality of life, thereby informing the care and support provided to mental health service users. In the case of BEL, it is possible that the life-and-health aspect of quality of life benefits more from the intervention, as participants tend to show greater changes in meaning-making and self-acceptance than in their actual life circumstances [6]. The study was guided by the following research questions:

- Did the group receiving the BEL intervention and the group receiving CAU differ in their improvements on two quality of life aspects—a personalized life-and-health aspect and a satisfaction-with-living environment aspect—from baseline to post-intervention and a 6-month follow-up?
- How were changes in various aspects of everyday occupations related to quality of life improvements in the sample as a whole?

It was hypothesized that the group receiving the BEL intervention would show greater improvement than the CAU group in the life-and-health aspect of quality of life, and that no group differences would emerge for the environmental aspect. Furthermore, it was hypothesized that subjective perceptions of everyday occupations would be associated with the life-and-health aspect of quality of life, whereas actual doing would be associated with the environmental aspect.

## Methods

The original RCT on which this re-analysis builds [4] was approved by an Ethical Review Board and adhered to the CONSORT guidelines. It was based on cluster randomization and data were collected at three time points – at baseline (T1), after completed intervention (T2) and at a 6-month follow-up (T3).

### The Interventions

#### Balancing Everyday Life (BEL)

The BEL intervention, as provided in the current study, is a group- and activity-based program with 14 sessions spread over 16 weeks, where each session lasted for 1.5–2 hours. It is framed as a course and led by an occupational therapist who has specific training for providing the BEL intervention and uses the BEL manual [5, 11]. The first 12 sessions follow specific themes relating to everyday life, such as motivators in life, healthy living, working life, and social activities. Sessions target both subjective experiences related to occupations and environmental aspects that might affect the possibility to engage in occupations. Each session is built up by a brief educational section and discussion of the session theme. The participants analyze their situation regarding everyday occupations, set desired activity goals related to the session themes, and develop strategies for how to reach them. For example, in one session the participants are to reflect on social relationships and social roles, including the meaning and value that social activities hold for them, and how they would like to change social relationships or social patterns. The strategies developed through this process of self-reflection are subsequently tried in a home assignment completed after the session in a real-life context. Goals and strategies may be re-negotiated in a subsequent session, if need be, and a new home assignment can follow.

The ultimate goal with BEL is that participants, not only together with peers from the course but also on their own, will develop the ability to reflect on their activity situations and gain strategies for adapting them, and accordingly enhance their satisfaction with everyday occupations.

#### Care as Usual (CAU) – Standard Occupational Therapy

CAU was occupational therapy according to “best practice”. CAU was often organized as a group intervention using creative or artistic occupations, daily living skills, social skills, or handicrafts as therapeutic media. Some mental health services offered individual therapy only, but it was based on similar occupations. CAU was not limited to a specific number of sessions or weeks, but to align with the BEL program, T2 occurred after 16 weeks of CAU and T3 was 6 months after T2. The choice of CAU as the comparison condition was deliberate and aligns with recommendations for pragmatic trials evaluating complex interventions [12].

#### Intervention Similarities

The BEL and the CAU interventions were both activity-based and were provided by occupational therapists who were part of a team that offered a range of ordinary mental health interventions, such as cognitive-behavioral therapy, general counselling, and psychotropic medication.

### Sample Size

We strove to recruit 60 participants from each of the following conditions: (a) BEL participants from specialized psychiatry run by the county councils; (b) BEL participants from community-based municipal psychiatry; (c) comparison participants from specialized psychiatry; and (d) comparison participants from community-based psychiatry. In line with the study aim, which does not address the influence of care context, conditions (a) and (b) formed the BEL group and conditions (c) and d) the CAU group. Comparisons between specialized and community-based psychiatry were the topic for a related paper [13].

### Randomization

The settings were included during 2012–2015. On the basis of blocks of four units, two were randomized to the BEL intervention and two to the control condition. With every fourth included setting, four lots were drawn by a colleague at the department, allotting two settings to each intervention condition. This procedure ensured allocation concealment, seen as utmost important to prevent bias [14]. Since the cluster design did not allow for blinding, other actions were taken to counteract bias. In addition to allocation concealment, measures were taken to ensure that all participants were equally met and treated. Both groups received the same information letter, which did not indicate whether their treatment was a new method or CAU. Nor was treatment allocation specified to the research assistants who collected the data.

### Settings and Participants

**Fig. 1:**
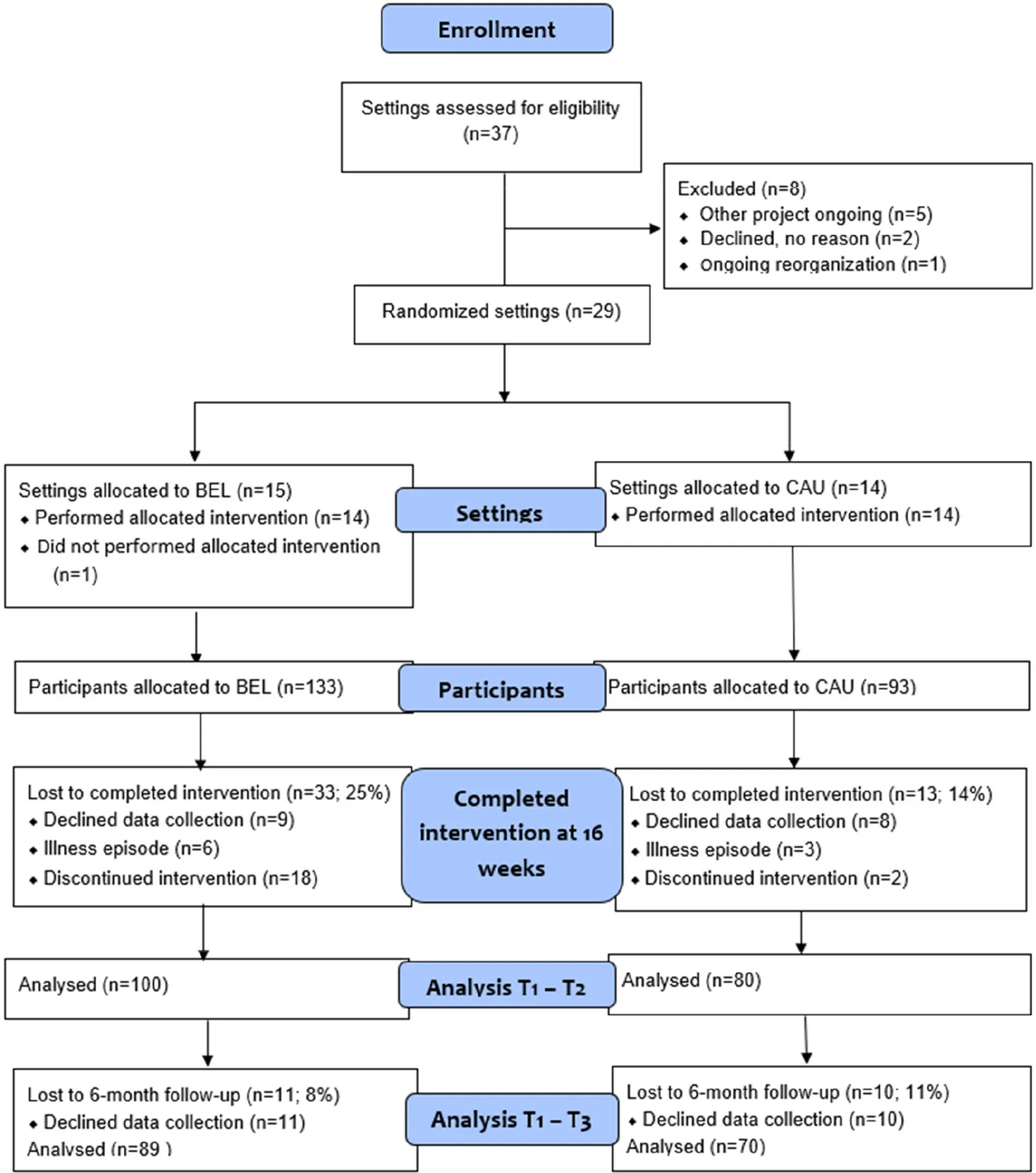
Flowchart of the RCT study

The process of selecting settings and participants is outlined in Figure 1. Initially, all outpatient units within general psychiatry, psychosis care, and community-based mental health services across three regions in Sweden were invited to join the study. However, eight settings were excluded due to ongoing projects or internal restructuring. Of the remaining 29 settings, 15 were randomly assigned to the BEL group and 14 to the CAU group. One BEL site later withdrew due to staff illness.

Each participating setting appointed an occupational therapist to act as a gatekeeper responsible for identifying eligible participants. To be included, individuals had to be aged 18–65 and diagnosed with a major mental health disorder. Accepted diagnoses included psychotic disorders, mood disorders, and neuropsychiatric conditions. Participants also needed to have sufficient proficiency in Swedish. Exclusion criteria included a primary diagnosis of substance use disorder or co-occurring dementia or developmental disorders.

Eligible individuals were invited to participate and received both verbal and written information from the gatekeeper. Those who agreed were contacted by a research assistant to schedule data collection. Data collection was conducted in a private, quiet room at each site, starting with participants signing a written informed consent form. Participants received a small amount of financial compensation, corresponding to 10 Euro, and reimbursement for travel expenses.

A total of 226 individuals were enrolled, 133 from BEL sites and 93 from CAU sites. Approximately 90% of participants in both groups were born in Sweden. Table 1 provides further details, showing that participants were similar across both groups in terms of sociodemographic and clinical characteristics.

**Table 1.** Sociodemographic and clinical characteristics of the participants

Characteristics	BEL group N=133	CAU group N=93	Chi2/ ***T-value***	P-value
Gender (% women)	77	67	3.11	Ns.
Age (mean, SD)	40 (11)	40 (11)	*−0.60*	Ns.
Living with partner (%)	30	26	3.39	Ns.
Highest educational level (%) Nine-year compulsory school or lower High school College/university	18 59 23	21 60 19	2.24	Ns.
Self-reported first diagnosis (%) Psychosis Anxiety/bipolar/depressive disorders ADHD/ADD/Aspergers Other	19 52 23 6	24 50 16 10	2.84	Ns.

Between the first (T1) and second (T2) data collection points, there were 33 dropouts (25%) in the BEL group and 13 (14%) in the CAU group, a statistically significant difference (p = 0.047). Most BEL group dropouts did not complete the intervention, often attending only one session. Dropout due to unwillingness to complete data collection or experiencing an illness episode occurred in both groups at similar rates. No significant differences were found between dropouts and completers on any of the variables listed in Table 1 (p = 0.155–0.712). The dropout rate between T2 and T3 did not differ between the BEL group and the CAU group (p = 0.527).

### Instruments

Self-administered and interview-based questionnaires were used, as specified below.

#### Two-Dimensional Assessment of Quality of Life

The self-administered Manchester Short Assessment of Quality of Life (MANSA) [7] includes 12 questions addressing satisfaction with life. One item focuses on life as a whole and 11 target different listed life areas. The ratings from these questions, which use a 7-point scale ranging from 1 = “could not be worse” to 7 = “could not be better”, are originally meant to be summarized into a quality of life score. However, the current re-analysis proceeded from previous research suggesting that quality of life as measured by MANSA is best understood as composed of two dimensions, where one concerns personal life and health and the other has environmental connotations [2, 3]. Those two studies arrived at two very similar but not identical quality of life dimensions, and since the Petkari et al. [2] study is based on a very large sample we chose their solution, where satisfaction with (1) Life as a whole, (2) Job/Being unemployed, (3) Financial situation, (4) Friendships, (5) Sex life, (6) Leisure activities, (11) Physical health, and (12) Mental health form the personal life and health dimension. Satisfaction with (7) Accommodation, (8) People one is living with/living alone, (9) Personal safety, and (10) Relationships with family form the environmental aspect. A Swedish MANSA version was used, found to have satisfactory properties in terms of adequate construct validity and good internal consistency [15]. Based on the current sample, Cronbach’s alpha (CITC range) for the two factors identified by Petkari et al. [2] was 0.80 (0.33–0.74) for the life and health aspect and 0.41 (0.20–0.25) for the environmental aspect.

#### Instruments assessing everyday occupations

Instruments were chosen that address both actual doing – the performance of occupations and the subjective experience the performance brings to the doer, in terms of engagement, satisfaction and value. Actual doing and subjective experiences may be seen as the end poles of a continuum of occupations where some can be classified near the extremes but others fall somewhere in between [16], as specified below.

#### The Role of Being a Worker

The Worker Role Self-assessment (WRS) [17, 18] is a self-administered questionnaire that assesses the respondent’s employment situation through statements targeting two aspects of the worker role: being a worker role holder and having the resources needed for that role. The instrument has demonstrated good construct and known-groups validity, as well as satisfactory internal consistency [18]. The 16-item WRS was used. Sample items for the worker role holder aspect are “I take a great deal of responsibility for the tasks I get” and “I have goals I want to attain in relation to work”. Examples of items addressing worker role resources are “have good habits and routines in my everyday life” and “I think I would be able to develop good routines in a future work”. The response scale has five alternatives, from 1 = “totally disagree” to 5 = “totally agree”. A higher rating indicates stronger beliefs/resources. Being a worker role holder is considered to reflect actual doing and resources for having a worker role to be a bit closer to the individual’s subjective perception of their ability to work in the future.

#### Occupational Engagement

The Swedish nine-item Profiles of Occupational Engagement in people with Severe Mental Illness (POES), shown to have good psychometric properties in terms of inter-rater agreement and construct validity [19–21], was used to address occupational engagement. In POES, occupational engagement is seen as a process involving external/objective aspects and internal/subjective aspects of occupational performance, including both anticipation and comprehension regarding the same [21]. Occupational performance refers to the accomplishment of meaningful occupations through the interplay among the person, their environment, and the occupation (American Occupational Therapy Association [22]. When administering the original POES, the rater is a professional, but in the current study a self-report version was used. Regardless of rater perspective, POES consists of a diary that covers the past 24 hours. The diary sheet has four columns; for the activity performed, the social context, the geographical context and reflections/feelings. Based on the diary content, the respondents rate their engagement in occupations according to nine items addressing, for example, balance between rest and activity, being able to move between places, and taking initiatives, etc. Four response alternatives are used, ranging from less (=1) to more (=4) engaged in activity. The ratings for the nine items are summed up, and a higher score indicates more occupational engagement. No psychometric study has as yet been performed on the self-report 24-hour version, but a self-report POES version limited to the productive hours of the day has shown good internal consistency, and a logical pattern of associations with occupational and functioning variables indicated construct validity [23]. Since occupational engagement is contingent on both what the respondent actually does (the first column of the diary sheet), the context (columns 2 and 3) and how the respondent perceives the doing (column 4), the engagement rating that follows is regarded a mix between actual doing and subjective perceptions of everyday occupations [16].

#### Activity Level and Satisfaction with Everyday Occupations

*Activity level* was estimated with the interview-based 13-item Satisfaction with Daily Occupations (SDO) instrument [24]. The research assistant posed the questions, but the respondent’s own ratings are registered. Each item has two parts, targeting common occupations of everyday life, covering the occupational domains of work, leisure, home and household maintenance, and self-care. The first part of an item concerns whether the respondent presently performs the occupation or not (yes=1, no=0). Affirmative responses are summed into an activity level score, reflecting actual doing.

*Satisfaction with everyday occupations* is an index based on the second part of the SDO items and constitutes a subjective perception of one’s everyday occupations. It is defined as doing valued things that align with one’s motivations and needs [24]. The respondents rate their satisfaction with having/not having the listed occupations on their present occupational repertoire, also if they do not currently engage in the activity in question. The SDO has shown very good internal consistency and adequate construct validity and test-retest stability [24–26].

#### Occupational Value

Occupational value, understood as a subjective perception of everyday occupations, was measured using the Swedish version of the self-report instrument Occupational Value with pre-defined items (OVal-pd) [27, 28]. It is a self-report instrument consisting of 18 items, formulated as statements. Six of the items feature concrete value, four socio-symbolic value, and eight self-rewarding value. Each item starts with the phrase “I feel that I have done things in the last month …”. Then each item addresses a specific occupational value experience, formulated as a statement. Some examples are as follows: “…because it was necessary to do those things” (concrete value), “…where I was in touch with other people” (socio-symbolic value) and “…because it was a true pleasure to do them” (self-rewarding value). The rating concerns the frequency of the occupational value experience. A 4-point scale is used, from “very rarely or never” (=1) to “very often” (=4). A higher score indicates more frequent occupational value experiences.

### Data Analysis

Tests for skewness and kurtosis indicated normal distribution for all variables and parametric tests were chosen. Change scores were calculated as the difference between the later measurement—completed intervention (T2 and T3, respectively)—and the baseline measurement (T1). Thus, a positive value indicates amelioration and a negative one indicates deterioration. Differences between the two intervention groups at T2 and T3, based on change scores from baseline, were estimated by independent samples t-test. Effect sizes were calculated according to Cohen’s *d*, and proposed limits for the magnitude of effect sizes are 0.20= small effect, 0.50= medium effect, and 0.80=large effect [29]. For categorical data, such as sex and education, the Chi^2^ test was used. Associations between variables were calculated by Pearson correlations and were based on the sample as a whole. Limits for interpreting the strength of correlations were set as follows: “very strong” for r ≥ 0.90, “strong” for 0.70 > r < 0.89, “moderate” for 0.40 > r < 0.69, “weak” for 0.10 > r < 0.39, and “negligible” for r < 0.10 [30]. In case of internal missing data, imputations with the individuals’ mean were made if they had responded to ≥ 75% of the items of an instrument. A p-value of <0.5 was set to signify statistical significance and the software used was the IBM SPSS version 31 [31].

## Results

### Quality of Life Improvements

The quality of life change scores for the two groups at completed intervention and at the 6-month follow-up are shown in Table 2. All values are positive, pointing to improvements. Regarding the life and health aspect, the BEL group had improved more than the CAU group at completed intervention, and the difference was statistically significant. Also at the follow-up, the BEL group improved more regarding the life and health aspect, and the p-value was exactly on the border of statistical significance. Regarding the environmental aspect, the differences were smaller and not statistically significant. Most effect sizes were small, but the one for change between baseline to the follow-up was moderate in size for the BEL group.

**Table 2.** Quality- of-life changes in the two groups

	Change from baseline to completed intervention (N=180)		T-value	P-value	Change from baseline to the six-month follow-up (N= 159)		T-value	P-value
	BEL group	CAU group			BEL group	CAU group		
Quality of life: the personalized life-and-health aspect	2.6 (7.5) ES=0.39	0.4 (6.6) ES=0.22	2.30	0.023	4 (8.3) ES=0.58	1.6 (7.2) ES=0.2	1.98	0.05
Quality of life: the satisfaction with living environment aspect	0.9 (4) ES=0.32	−0.2 (3.6) ES=0.09	1.93	0.055	0.7 (4.3) ES=0.25	0.5 (4) ES=0.18	3.44	0.731

*ES*, effect size

Correlations between the two types of quality of life change scores, based on the total sample, were r=0.34 (p<0.001) for the change scores between baseline and completed intervention and r=0.51 (p<0.001) for change scores between baseline and follow-up.

### Associations Between Change in Occupational Factors and Quality of Life Change

Associations between change scores for various aspects of everyday occupations and quality of life are displayed in Table 3. Changes in the variables of worker role resource and occupational engagement, regarded as situated in the middle of the actual-doing-and-subjective-perceptions continuum, as well as satisfaction with everyday occupations and occupational value, regarded as subjective perceptions, were all associated with change in both quality of life aspects at completed intervention and at the 6-month follow-up. The correlations were in the realm of moderate to weak, ranging between r=0.29 and r=0.46 for the life-and-health aspect of quality of life and between r=0.17 and r=0.31 for the environmental aspect. Change towards increasingly being a worker role holder was weakly associated with an increase in the life-and-health aspect of quality of life on both occasions and with the environmental aspect at completed intervention. Increased activity level was related to improved quality of life in terms of life and health at the follow-up, but not at completed intervention; nor was increased activity level related to the environmental quality of life aspect at any measurement point.

**Table 3:** Correlations between changes in various aspects of everyday occupations and quality of life, based on the sample as a whole

	Quality of life change: the personalized life-and-health aspect		Quality of life change: the satisfaction with living environment aspect	
	Baseline to completion (N=180)	Baseline to follow-up (N=159)	Baseline to completion (N=180)	Baseline to follow-up (N=159)
Activity level change	r=0.08 p=0.30	r=0.25 p=0.001	r=0.001 p=0.99	r=0.11 p=0.166
Worker role holder change	r=0.28 p<0.001	r=0.20 p=0.01	r=0.16 p=0.037	r=0.08 p=0.324
Resources for worker role change	r=0.30 p<0.001	r=0.37 p<0.001	r=0.21 p=0.004	r=0.20 p=0.012
Occupational engagement change	r=0.29 p<0.001	r=0.46 p<0.001	r=0.23 p=0.007	r=0.24 p=0.005
Satisfaction with everyday occupations change	r=0.41 p<0.001	r=0.39 p<0.001	r=0.17 p=0.022	r=0.25 p=0.002
Occupational value change	r=0.41 p<0.001	r=0.37 p<0.001	r=0.31 p<.001	r=0.26 p=0.001

Sizes of correlation coefficients were interpreted as “moderate” for 0.40 > r < 0.69, “weak” for 0.10 > r < 0.39, and “negligible” for r < 0.10 [30]

## Discussion

Discerning two dimensions of quality of life as assessed by MANSA revealed interesting results. The first hypothesis was confirmed; the BEL group improved more than the CAU group regarding the life-and-health aspect of quality of life, particularly at completed intervention, but not regarding the environmental aspect. The previous BEL paper used only the summed score and did not detect a difference in quality of life improvement at completion of the intervention, although both groups then scored higher compared to baseline [4]. The current findings underscore the importance of viewing quality of life as a multidimensional phenomenon, as proposed in prior research [2, 3], while also adding to the benefits of the BEL intervention compared to CAU.

Although the level of engagement in everyday occupations is often reported to be low among mental health service users, such occupations are already present in their repertoire of everyday doings [32, 33]. Everyday occupations may therefore be something to build further on as a tentative channel for support towards quality of life improvements and personal recovery, which makes knowledge about associations between everyday occupations and quality of life of considerable scholarly and clinical interest. The hypothesis regarding associations between change scores on occupational factors and quality of life changes was partly verified. Increases in the subjective perceptions of everyday occupations, also the ones that were regarded as situated on the middle of the continuum, were associated with improvements in the life-and-health quality of life aspect. However, subjective perceptions were also associated with change in the environmental quality of life aspect, although those correlations seemed somewhat weaker. Bearing in mind that the alpha value for the environmental aspect was low, this result should also be interpreted with caution. Furthermore, the findings based on the two occupational aspects regarded as strictly actual doing – activity level and being a worker role holder – deviated from the hypothesis and showed weak and often non-significant associations with both quality of life aspects on both occasions.

Previous studies on relationships between everyday occupations and quality of life have not discerned dimensions of quality of life, nor have they focused on associations between change scores in mental health intervention contexts. For example, the intervention action over inertia, an intervention somewhat similar to the BEL intervention, has not been evaluated using quality of life measures but rather outcomes such as occupational engagement and recovery [34, 35]. This makes comparisons with the current findings difficult, but other evaluations of activity-based interventions, using unidimensional quality of life as the outcome measure, may offer some comparability. The result picture from such evaluation studies is scattered, with some interventions found effective in relation to quality of life [4, 36, 37] and others not [38]. The current study adds to previous research by discerning clear and consistent associations between increases in subjective experiences of everyday occupations and quality of life, particularly the life-and-health aspect of quality of life. Thus, activity-based interventions that stress subjective experiences may be effective in relation to quality of life improvements, first and foremost not only the life and health aspect but also the environmental aspect. The latter contains many different elements, such as secure housing, perceived safety, supportive social relationships, and opportunities for community participation, which may all substantially affect service users’ quality of life.

The environmental dimension of social interactions is often—although not necessarily—embedded in everyday occupations. Interestingly, people with severe mental illness have reported everyday occupations and social interaction as vital for their sense of meaningfulness in life [39]. It is therefore possible that everyday occupations with inbuilt social interaction, such as group activities, study circles, and meeting points (all providing opportunities of informal peer support which might combat feelings of stigma), would be the most effective activity-based form of intervention for quality of life support. Co-production and ensuring service users genuine involvement when developing an intervention would also play a role regarding effectiveness. Besides advancing occupational justice, it would also facilitate more accurate assessments and the development of tailored interventions [40], in which relevant environmental factors and social issues can be incorporated. In fact, the successful interventions mentioned above [4, 36, 37] were all based on combinations of everyday occupations and social interaction, and most were also co-produced. Future research should address whether combining everyday occupations and social interaction is more successful than either component alone for improvements in different quality of life domains.

### Methodological Considerations

This study was based on a solid data set from a carefully designed RCT [4], which still had some limitations. Using a cluster RCT design ensured that the settings were allocated in an unsystematic manner to the interventions, but it did not allow for blinding. Measures taken to counteract bias included stipulating strict criteria for participant selection, inviting all eligible prospective participants, providing both intervention groups with identical information, and maintaining allocation concealment vis-à-vis the project assistants who collected the data.

The fact that the comparison condition was not standardized in duration or content may have entailed variability in treatment exposure and attenuated between-group differences. This limits the precision with which the intervention effect can be estimated, but CAU is commonly used to reflect the care participants would receive in routine clinical practice, thereby allowing an assessment of the intervention’s effectiveness under real-world conditions rather than under tightly controlled experimental circumstances. This is particularly relevant when the research question concerns whether the intervention provides added value beyond current standard practice. Another point worth mentioning is that completing the POES requires participants to reflect on their everyday occupational situation, which may have influenced the manipulation. As a result, differences between the groups may have been reduced, potentially underestimating the effect of the BEL intervention.

Furthermore, the dropout rate was higher in the BEL group from T1 to T2. This suggests that the BEL intervention involved some form of difficulty for participants that was not experienced in the CAU group. It may be that BEL required greater investment of time and/or energy, or that it was simply unfamiliar and therefore more demanding. In any case, the difference in dropout rates weakens the internal validity of the study.

As reported in the Methods section, the subscale addressing the environmental aspect of quality of life showed poor internal consistency. This may partly reflect the small number of items included in this subscale (only four). Nevertheless, the findings related to the environmental aspect should be interpreted with caution. For future research using a two-factor structure of the MANSA, it would be advisable to expand the instrument with additional items targeting environmental quality of life aspects, or possibly develop an assessment specifically designed for this purpose.

To our knowledge, this is the first of its kind to explore relations between activity change and quality of life change among mental health service users. It needs to be replicated before any safe conclusions can be made, but the findings can be used to set up hypotheses for future intervention research, as exemplified above, but also for other longitudinal research. Epidemiological research in the general population and studies performed with other target groups than mental health service users are possible avenues for that.

One of the methodological drawbacks in the re-analysis is that the occupational measures were not initially chosen to match the two MANSA dimensions, but only to address various aspects of everyday occupations. Obviously, those measures supposed to reflect actual doing did not harmonize optimally with the environmental aspect of quality of life. However, this may also reflect an artifact arising from the low homogeneity of the environmental scale, although being a worker role holder showed expected, although weak, associations with environmental quality of life. Activity level may still have been too indistinct a measure. It reflects only the number of activities a person engages in, not the degree of involvement, effort invested, time spent, or similar qualitative aspects. These parameters should be included in future attempts to replicate the current study.

Only two dimensions of quality of life were in focus in the current study, which may be seen as both an advantage and a drawback. Since several aspects of everyday occupations were addressed, it was advantageous to concentrate on two major dimensions of quality of life in this first study investigating associations between occupational changes and quality of life changes. In that, it was possible to obtain a fairly clearcut result to be used for further hypotheses. Several quality of life dimensions would have yielded a more split pattern of findings, perhaps not indicating where to go next. But the restricted number of dimensions might also be seen as a disadvantage since quality of life is a complex phenomenon with many facets [41].

Finally, the data analyzed here were collected approximately ten years ago, and the timeliness of the current paper may therefore be questioned. However, the debate concerning the two quality of life aspects in focus has gained momentum more recently, particularly following the publication by Petkari et al. in 2020. We consider the BEL material sufficiently unique to warrant re-examination and publication, as demonstrated by the new insights it has generated.

Notwithstanding the limitations discussed above, the study can be considered to possess sufficient internal and external validity to allow cautious generalization to similar settings and service-user populations.

## Conclusion

The main conclusions from this study are twofold:

- A two-dimensional perspective revealed significant quality of life improvements that had gone undetected in an earlier RCT of the BEL, when an overall score was used.
- Satisfaction with occupations was more closely interrelated with quality of life than was actual doing, and should hence be prioritized in occupational therapy practice.

A multidimensional perspective on quality of life is thus recommended in future intervention research and in clinical practice. Particularly, the environment—pivotal in occupational therapy practice—must not be neglected. The relationships revealed between changes in everyday occupations and quality of life developments strengthen the picture obtained from previous intervention research that the subjective perceptions of everyday occupations are key in support towards improved quality of life and increased personal recovery. However, the results from the current study are not straightforward and much remains to be known regarding how everyday occupations can be utilized in the support of mental health service users towards better quality of life.

## Data Availability

The data sets analyzed during the current study are not publicly available due to the restriction set by the Swedish Act concerning the Ethical Review of Research Involving Humans but are available from the corresponding author on reasonable request.
